# Cerebrospinal Fluid and Plasma Small Extracellular Vesicles and miRNAs as Biomarkers for Prion Diseases

**DOI:** 10.3390/ijms22136822

**Published:** 2021-06-25

**Authors:** Óscar López-Pérez, David Sanz-Rubio, Adelaida Hernaiz, Marina Betancor, Alicia Otero, Joaquín Castilla, Olivier Andréoletti, Juan José Badiola, Pilar Zaragoza, Rosa Bolea, Janne M. Toivonen, Inmaculada Martín-Burriel

**Affiliations:** 1 Laboratorio de Genética Bioquímica (LAGENBIO), Instituto Agroalimentario de Aragón (IA2), Instituto de Investigación Sanitaria de Aragón (IISAragón), Universidad de Zaragoza, Miguel Servet 177, 50013 Zaragoza, Spain; oscarlzpz@gmail.com (Ó.L.-P.); davidsanzrubio91@gmail.com (D.S.-R.); ahernaiz@unizar.es (A.H.); pilarzar@unizar.es (P.Z.); toivonen@unizar.es (J.M.T.); 2 Centro de Investigación en Encefalopatías y Enfermedades Transmisibles Emergentes, Instituto Agroa-Limentario de Aragón (IA2), Instituto de Investigación Sanitaria de Aragón (IISAragón), Universidad de Zaragoza, Miguel Servet 177, 50013 Zaragoza, Spain; mbetancorcaro@gmail.com (M.B.); aliciaogar@unizar.es (A.O.); badiola@unizar.es (J.J.B.); rbolea@unizar.es (R.B.); 3 Centro de Investigación Biomédica en Red de Enfermedades Neurodegenerativas (CIBERNED), Instituto de Salud Carlos III, 28031 Madrid, Spain; 4 Translational Research Unit, Hospital Universitario Miguel Servet, Instituto de Investigación Sanitaria de Aragón (IISAragón), 50009 Zaragoza, Spain; 5 CIC bioGUNE and IKERBASQUE, Basque Foundation for Science, 48230 Bizkaia, Spain; jcastilla@cicbiogune.es; 6 UMR Institut National de la Recherche Agronomique (INRA)/École Nationale Vétérinaire de Toulouse (ENVT) 1225, Interactions Hôtes Agents Pathogènes, Ecole Nationale Vétérinaire de Toulouse, 31000 Toulouse, France; o.andreoletti@envt.fr

**Keywords:** extracellular vesicle, prion, PMCA, cerebrospinal fluid, plasma, bioassay

## Abstract

Diagnosis of transmissible spongiform encephalopathies (TSEs), or prion diseases, is based on the detection of proteinase K (PK)-resistant PrP^Sc^ in post-mortem tissues as indication of infection and disease. Since PrP^Sc^ detection is not considered a reliable method for in vivo diagnosis in most TSEs, it is of crucial importance to identify an alternative source of biomarkers to provide useful alternatives for current diagnostic methodology. Ovine scrapie is the prototype of TSEs and has been known for a long time. Using this natural model of TSE, we investigated the presence of PrP^Sc^ in exosomes derived from plasma and cerebrospinal fluid (CSF) by protein misfolding cyclic amplification (PMCA) and the levels of candidate microRNAs (miRNAs) by quantitative PCR (qPCR). Significant scrapie-associated increase was found for miR-21-5p in plasma-derived but not in CSF-derived exosomes. However, miR-342-3p, miR-146a-5p, miR-128-3p and miR-21-5p displayed higher levels in total CSF from scrapie-infected sheep. The analysis of overexpressed miRNAs in this biofluid, together with plasma exosomal miR-21-5p, could help in scrapie diagnosis once the presence of the disease is suspected. In addition, we found the presence of PrP^Sc^ in most CSF-derived exosomes from clinically affected sheep, which may facilitate in vivo diagnosis of prion diseases, at least during the clinical stage.

## 1. Introduction

Transmissible spongiform encephalopathies (TSEs) are fatal neurodegenerative diseases that affect humans and animals [1]. TSEs result from misfolding of a native cellular prion protein (PrP^C^) to an infectious and pathological conformation (PrP^Sc^) in a self-propagating reaction [2]. Ovine and caprine scrapie was the first TSE described and is considered the prototype of these diseases [3]. As in other TSEs, like bovine spongiform encephalopathy in cattle or variant Creutzfeldt-Jakob disease (vCJD) and Kuru in humans, in natural scrapie, prions enter the body through infection of the gastrointestinal tract, then, accumulate in the lymphoreticular system and access the enteric nervous system (ENS) that seems to be the entry to neural tissues [4]. To date, the precise mechanisms that explain prion spread to the central nervous system (CNS) are still not well known.

Post-mortem detection of PrP^Sc^ in the brain tissue of diseased individuals is used as a biochemical marker for the presence of infection and disease [5]. However, the use of this protein as an effective diagnostic biomarker is not considered a reliable method for in vivo diagnosis since the presence of PrP^Sc^ is minimal in accessible tissues and body fluids such as blood or urine [6]. In addition, new protease-sensitive PrP^Sc^ isoforms have been discovered that are not detectable by current diagnostic methods [7,8]. Therefore, it is necessary to identify biomarkers other than PrP^Sc^ that can be detected from accessible tissues or fluids to provide useful alternatives for current diagnostic methodology.

Exosomes are small vesicles surrounded by membrane with a variable size ranging from 30 to 150 nm [9]. These particles are secreted by cells and have been found in body fluids such as urine, breast milk, saliva, blood or cerebrospinal fluid (CSF) [9]. Exosomes, originated from endosomes, are released by cells and can reprogram surrounding or distant cells after selective taking up [10]. The exosomal “cargo”, the vesicular content of proteins, lipids, non-coding RNAs, mRNAs and microRNAs (miRNAs), can be different according to the exosome origin. They contain membrane transport and fusion proteins, tetraspanins and heat-shock proteins that are used as positive markers for exosome characterisation [11]. 

In vitro studies have demonstrated the release of PrP^C^ and infectious PrP^Sc^ by different types of infected cells in association with exosomes [12,13,14,15], suggesting a potential role of these particles in intercellular transmission, prion spreading and neuroinvasion, although different prion strains might use the exosomal pathway with different efficiencies [16]. The release of exosomes in prion diseases seems to be related with the autophagic process. Blocking autophagy in prion infected neuronal cell cultures boosts release of prions in exosomes and increases intercellular spread of prion infection [17]. Autophagy process is impaired in clinical scrapie [18], so this alteration could promote exosome production and release to biofluids in vivo.

The presence of PrP^C^ among the protein cargo of blood circulating exosomes has been demonstrated in humans [19] and sheep [20]. PrP^Sc^ was first detected by protein misfolding cyclic amplification (PMCA) in extracellular vesicles (EV) isolated from blood plasma of mice infected with mouse-adapted vCJD [21] and from 263K scrapie-infected hamsters [22]. Besides the demonstration of infectivity of exosomes isolated from these prion-inoculated murine models, to our knowledge, the potential of extracellular vesicles to mediate pathological prion infectivity has never been described in the natural disease, even though attempts to detect the presence of the PrP^Sc^ in plasma-derived exosomes have been carried out in natural scrapie sheep using Western blot [20]. More sensitive techniques such as PMCA or murine bioassays could elucidate if plasma exosomes harbour aberrant prion protein in natural scrapie. 

Brain pathophysiology is reflected in CSF and the presence of EV in this biofluid represents a potential source of biomarkers for neurological diseases [23]. The levels of PrP^C^ in CSF have been considered to be a potential biomarker for several neurodegenerative diseases [24], including ovine scrapie [25], as its concentration seems to decrease in CJD affected individuals [26,27]. It has been reported that ovine CSF-derived exosomes are enriched in PrP^C^ [28] but, to our knowledge the presence of PrP^Sc^ in EV from CSF has not been previously described. 

The interest of exosomes in TSEs is not limited to the determination of PrP^Sc^, which could facilitate diagnosis and be crucial for prion transmission. Proteins and microRNAs (miRNAs) are molecular constituents of exosomes and promising biomarkers for many diseases, including neurodegenerative diseases [29,30]. Exosomes released in vitro by prion-infected neuronal cells have altered miRNAs profiles compared to non-infected exosomes [31]. In a previous work, we demonstrated the alteration of circulating miRNAs in plasma of scrapie-infected sheep [32]. In this study, elevated levels of miR-342-3p and miR-21-5p were found in plasma. However, if these potential biomarkers of prion disease are free in plasma or included in the exosomal fraction is still unknown. 

The relevance of exosomes in prion diseases has only been analysed in vitro and in murine models of TSE. The aim of this work was to analyse exosomes from blood and CSF in sheep naturally infected with scrapie, as an animal model of prion diseases. First, we evaluated the presence of PrP^Sc^ (proteinase K-resistant abnormal PrP, also known as PrP^res^) by PMCA, which would facilitate in vivo diagnosis. Then, their infectivity or ability to spread disease in the CNS was evaluated by bioassay. Finally, as the miRNA profile in exosomes could change with the pathological status of the individual, the potential of exosomes as a source of biomarkers was evaluated analysing the levels of candidate miRNAs described to be altered in different prion diseases and their models. These included miR-342 [31,32,33,34], miR-21 [31,34], miRNAs of let-7 family [15,31,35], miR-146a [31,34,35,36,37], miR-494 [33] and miR-128 [31,38]. 

## 2. Results

### 2.1. Exosome Isolation and Characterisation

**Figure 1:**
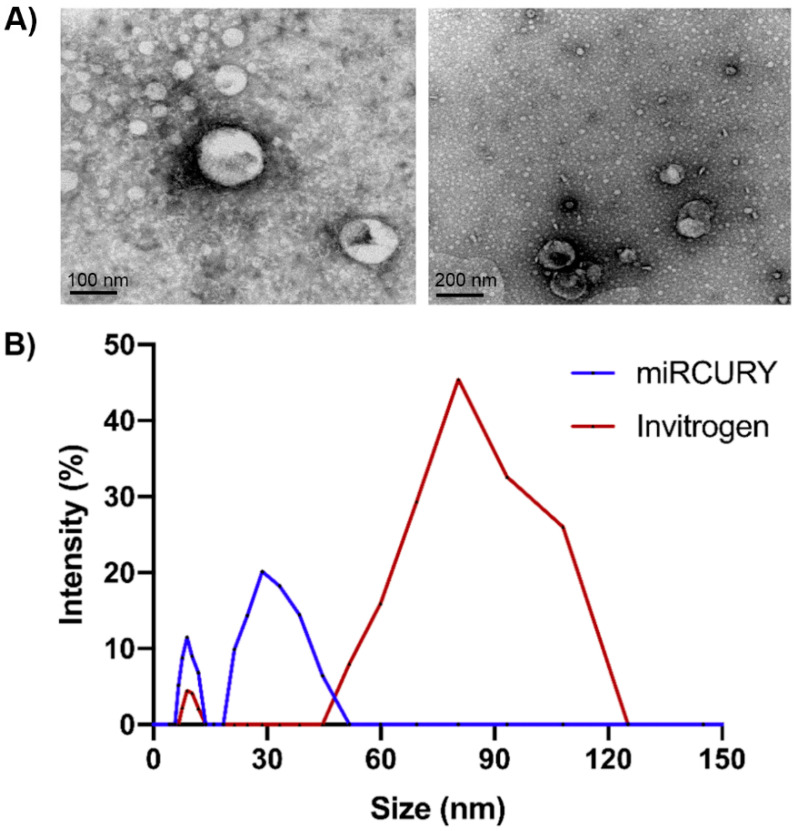
(**A**) Characterisation of sEVs derived from cerebrospinal fluid by transmission electron microscopy (TEM). Images show the presence of small spheres smaller than 200 nm, obtained with Invitrogen isolation kit (left, 100 nm bar) and Exiqon isolation kit (right, 200 nm bar). (**B**) Characterisation of sEV derived from blood plasma by dynamic light scattering (DLS). Graph shows that vesicles isolated from plasma displayed a mean diameter ranging between 30 and 120 nm. Intensity profiles showed that the major sEV population was approximately 50–120 nm in diameter (Invitrogen kit) and a second minor population was approximately 20–50 nm (Exiqon miRCURY kit).

We successfully isolated exosomes from plasma and CSF using two commercial kits (miRCURY^TM^ Exosome Isolation Kit (Exiqon, Düsseldorf, Germany) and Total Exosome Isolation Kit (Invitrogen, Waltham, MA, USA)). Exosomes were visualised using transmission electron microscopy (TEM). Figure 1A shows the presence of small spheres with diameters between 30 and 120 nm in samples obtained from CSF, using both isolation kits. According to the MISEV2018 guidelines [39], hereafter we will refer to these vesicles smaller than 200 nm as small extracellular vesicles (sEVs).

Consistently, dynamic light scattering (DLS) assays showed that vesicles isolated from plasma displayed a mean diameter ranging between 30 and 120 nm (Figure 1B). DLS intensity profiles showed that the major sEV population obtained with Invitrogen kit was approximately 50–120 nm in diameter. Size of the particles obtained with Exiqon miRCURY kit was smaller, with a peak in approximately 30 nm. The amount of sEVs isolated from CSF was not sufficiently high for the analysis by DLS.

### 2.2. Detection of PrP^res^ in sEVs by PMCA

PMCA allows amplification of minimal amounts of pathological prion protein. We performed this technique with isolated sEVs using brain homogenate from Tg338 transgenic mice expressing the ovine VRQ prion allele as a substrate [40]. A total of 24 sEV samples derived from plasma and obtained with Invitrogen kit were analysed by PMCA. After four successive rounds of PMCA, no proteinase K (PK)-resistant abnormal PrP could be detected by Dot blot or Western blot in any sample, whereas the brain homogenate from scrapie sheep included on each run to check the amplification performance displayed positivity in reactions seeded with a 10^−7^–10^−8^ dilution, suggesting that the presence of PrP^res^ in plasma-derived sEVs, if any, is at least 8 log10-fold lower than that detected in sheep brains (Figure S1).

**Figure 2:**
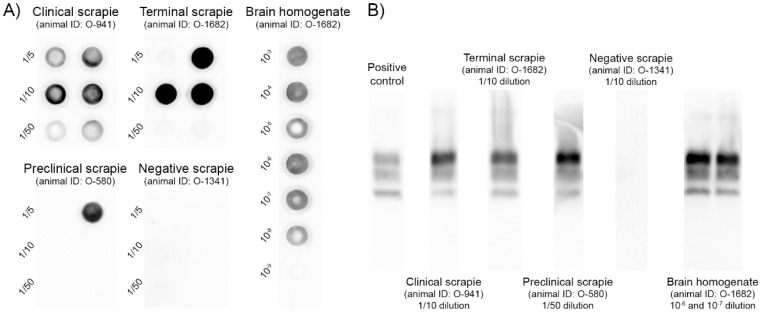
Detection of the pathologic prion protein (PrP^Sc^/PrP^res^) by Dot blot (**A**) and Western blot (**B**) in PMCA reactions seeded with serially diluted (1/5, 1/10 and 1/50) CSF-derived sEVs obtained from scrapie-affected sheep. Immunodetection was performed using the monoclonal Sha31 antibody. (**A**) Representative images of PrP^res^ detection by Dot blot after four PMCA rounds of CSF-derived sEVs from one sheep at clinical stage of scrapie, one sheep at terminal stage, one sheep at preclinical stage and one negative sheep. A brain homogenate from scrapie sheep (10^−3^ to 10^−9^ diluted) is also shown for comparison. Some dots were subjected to Western blot for PrP^res^ profile detection. (**B**) Representative images of PrP^res^ detection by Western blot after four PMCA rounds of CSF-derived sEVs from one sheep at clinical stage of scrapie (1/10 dilution), one sheep at terminal stage (1/10 dilution), one sheep at preclinical stage (1/50 dilution), one negative sheep (1/10 dilution) and a brain homogenate from scrapie sheep (10^−6^ and 10^−7^ dilution). A proteinase K-digested classical scrapie isolate (Dawson strain) was used as positive control.

On the contrary, after four rounds of PMCA, PrP^res^ was detected in reactions seeded with CSF-derived sEVs (obtained with Invitrogen kit) in six out of eight samples from clinically affected sheep and three out of three samples from sheep at terminal stage (Figure 2A and Figure S2). However, PrP^res^ was detected only in one out of six sEV samples obtained from CSF of preclinical scrapie sheep. No PrP^res^ signal was detected in reactions seeded with CSF-derived sEVs from the negative control sheep, confirming the specificity of the technique. The Western blot electrophoretic PrP^res^ pattern observed after PMCA amplification was indistinguishable from that observed in the brain homogenate from scrapie sheep, displaying a PrP^res^ molecular signature consistent with classical scrapie prions characterised by a three-banded pattern with a 21 kDa unglycosylated band (Figure 2B).

### 2.3. Evaluation of sEV Infectivity

CSF and CSF-derived sEVs from one negative control sheep (animal ID: O-1341) and two scrapie-infected sheep (animals ID: O-941 and O-1682) were also tested for infectivity by bioassay in Tg338 transgenic mice. A total of 27 mice were intracerebrally inoculated: nine mice with CSF-derived sEVs obtained with Exiqon kit, nine mice with CSF-derived sEVs obtained with Invitrogen kit and nine mice with total CSF. In each group, three mice were inoculated with CSF or CSF-derived sEVs from the negative control sheep and six mice with CSF or CSF-derived sEVs from the aforementioned two positive scrapie sheep.

Most animals were sacrificed by end point criteria (from 232 days post inoculation (dpi) of a mouse inoculated with scrapie CSF to 647 dpi of a mouse inoculated with negative CSF) or at the end of the study. Three of the mice inoculated with CSF-derived sEVs isolated with the Exiqon miRCURY kit died without showing symptoms compatible with scrapie or any other end point criteria at ages older than 381 dpi.

The post-mortem PET-blot analysis did not reveal any presence of PrP^Sc^ in any inoculated mice (Figure S4) and no significant differences were observed in haematoxyline-eosine staining tissue sections of mice inoculated with sEVs obtained from scrapie and control sheep. 

### 2.4. miRNA Profiles in Plasma-Derived sEVs

**Figure 3:**
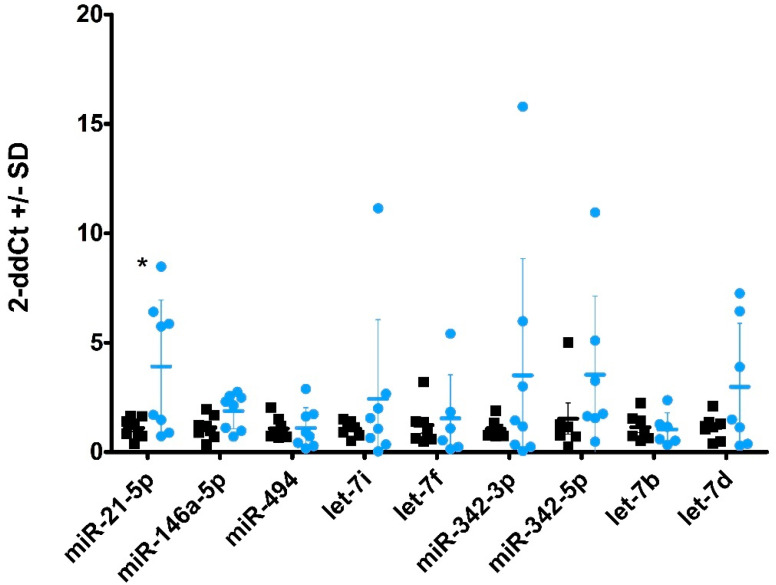
miRNA profiles in plasma-derived sEVs. Fold-change of miRNAs in sEVs derived from blood plasma of scrapie sheep (blue circles) compared with control sheep (black squares) as measured by qPCR. Nine miRNAs were found at detectable levels. Significantly increased levels were found for miR-21-5p (*p* < 0.05). Data are expressed as a relative expression value after 2^−ΔΔCt^ conversion using the mean of healthy control sheep as a calibrator, +/− standard deviation (SD). Statistical significance was assessed by Student’s *t*-test (* *p* < 0.05). The Exiqon kit was used for sEVs purification.

We recently described alterations in miRNA profile in plasma from scrapie-infected sheep [32]. In the present work, we analysed the same set of miRNAs in sEVs isolated from plasma of sheep naturally infected with scrapie. Nine miRNAs were successfully amplified in plasma sEVs by qPCR and, of these, miR-21-5p was present in a significantly higher level (*p* < 0.05) in sEVs obtained from scrapie-infected sheep (Figure 3). Additionally, miR-146a showed a tendency for upregulation but this did not reach statistical significance (*p* = 0.065). As a general observation, the dispersion between samples was greatly increased in scrapie sheep compared with controls.

### 2.5. miRNA Profiles in CSF and CSF-Derived sEVs

**Figure 4:**
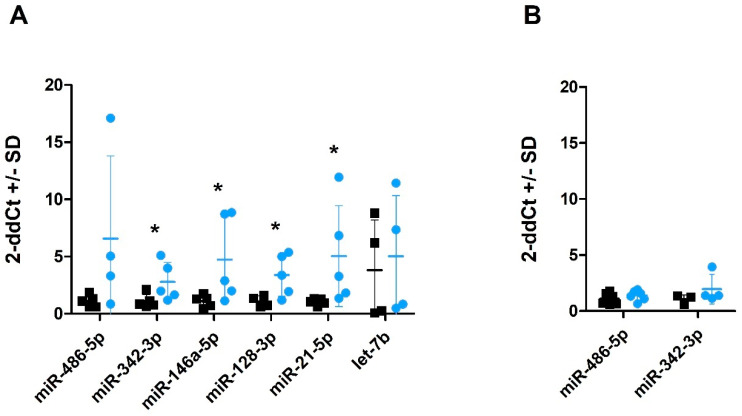
Quantification of miRNAs in CSF and CSF-derived sEVs by qPCR. Fold change of miRNAs in total CSF (**A**) and sEVs derived from CSF (**B**) of scrapie sheep (blue circles) compared with control sheep (black squares). Data are expressed as a relative expression value after 2^−ΔΔCt^ conversion using the mean of healthy control sheep as a calibrator, +/− standard deviation (SD). Statistical significance was assessed by Student’s *t*-test (* *p* < 0.05). sEVs were isolated using the Exiqon kit.

Six miRNAs were successfully amplified in CSF (Figure 4A) with a Ct value lower than 34, which was set as a limit for reliable detection. Four of these (i.e., miR-342-3p, miR-146a-5p, miR-128-3p and miR-21-5p) displayed a level significantly higher (*p* < 0.05) in CSF from scrapie-infected sheep compared to healthy control sheep (Figure 4A; see also Figure S3 for difference in Ct values between the control and scrapie samples presented in Figure 4A). Moreover, miR-132-3p was detected in four out of six scrapie CSF whereas it was only present in extremely low levels in one control (Figure S3). Levels of only two of the tested miRNAs were sufficient for quantification in sEVs derived from CSF. miR-486-5p was present in relatively high levels consistent with the previously published data suggesting exosomal enrichment of this miRNA [41]. miR-342-3p was present in low levels and not detectable in all samples (Figure 4B). No significant changes were detected in sEV miRNAs between scrapie and control samples.

## 3. Discussion

Exosomes are nanosized vesicles secreted by cells under physiological and pathological conditions. Their protein and nucleic acid content, or cargo, is altered in disease and have been postulated as a source of potential biomarkers in many diseases, including neurodegenerative diseases [42]. Detection of the pathognomonic biomarker PrP^Sc^ in biofluids is one of the strategies to help in the diagnosis of prion diseases. In vitro studies have suggested that PrP^Sc^ can be released from cells in exosome-infectious vesicles, providing a potential mechanism for the spread of infectivity between cells [12,43,44]. However, the mechanisms underlying PrP^Sc^ trafficking between and within neuronal cells are not well defined in vivo. Blood has been shown to contain disease-associated PrP^Sc^ in naturally and experimentally infected sheep [45,46,47,48], and in humans infected with vCJD [49,50]. Moreover, PrP^Sc^ has been detected in EV preparations containing exosomes from plasma of mice infected with mouse-adapted prions by PMCA, suggesting that exosomes may have a relevant role in carrying PrP^Sc^ in blood [51]. Here, the presence of the pathological form of the protein in plasma-derived sEVs has been investigated in the natural prion disease. We isolated sEVs using commercial kits and the presence of sEVs in the precipitate was confirmed by TEM. After four rounds of PMCA, no PrP^res^ could be detected in any sample of plasma derived sEVs, whereas the original brain homogenate displayed positivity in a 10^−7^ to 10^−8^ dilution, suggesting that the presence of PrP^Sc^ of plasma-derived sEVs, if any, is at least 8 log10-fold lower than that detected in sheep brains. Therefore, the detection of PrP^Sc^ by PMCA in these vesicles does not represent a useful tool for natural scrapie diagnosis. 

On the other hand, ovine CSF-derived sEVs seem to be enriched in PrP^C^ [28], but the presence of PrP^Sc^ in these vesicles has not been described. In this work, we detected the pathological form of the protein in CSF-derived sEVs after four rounds of amplification, in six out of eight samples from clinically affected sheep and three out of three samples from sheep at terminal stage. Unfortunately, PrP^Sc^ was detected only in one out of six sEV samples obtained from CSF of preclinical scrapie sheep, which limits its use for early diagnosis of prion diseases. Despite positive results from PMCA, infectivity of these particles was not observed in vivo. We used precipitation-based kits to isolate the sEVs, which could interfere the ability of PrP^Sc^ infection in vivo. On the other hand, we did not quantify the amount of the exact numbers of sEVs inoculated and a dose adjustment could be necessary for in vivo bioassays. 

Exosomes can serve as a source of biomarkers. MicroRNAs are small RNA molecules that produce a post-transcriptional regulation of target genes [52]. They are present in exosomal cargo and it seems that loading of miRNAs into exosomes is not a random process [53]. Altered miRNA profiles in CSF or blood exosomes associated with neurodegenerative disorders have revealed new biomarkers in the diagnosis of Alzheimer’s disease (AD) [30,54], amyotrophic lateral sclerosis [29] or Parkinson’s disease [55]. We investigated here the potential of circulating sEVs for the diagnosis of ovine scrapie.

miRNA profiles are changed in the CNS of different TSE models [36,38] and in brains from CJD patients [33,37]. In a previous work, we identified two altered miRNAs in plasma from sheep showing clinical signs of scrapie [32]. Here, we analysed in plasma-derived sEVs the same set of miRNAs that were previously reported to be altered in prion infected models. We did not observe any enrichment of miRNA levels in sEVs and only the amount of miR-21-5p differed between control and scrapie plasma. Exosome-shuttling miR-21 has been proposed as a potential biomarker for many diseases including a prognostic biomarker for oesophageal cancer patients [56], or even as a universal biomarker to identify cancers [57]. Due to its effects on cardiac contractility and calcium handling, exosomal miRNA secreted by mesenchymal stem cells have been proposed as possible therapy for future stem cell-based cardiotherapies [58]. However, neurotoxicity for exosomal miR-21-5p linked to simian immunodeficiency virus has also been proposed [59]. miR-21-5p is one of the most abundant miRNAs in several kinds of exosomes and the increase of this miRNA associated to different cancers could be linked to the increment in exosome production of cancer cells [60]. It could be plausible that the scrapie-associated increase observed here is also due to an increment in exosome release linked to the disease and not to specific packaging of this miRNA by scrapie infected or reactive cells. 

Changes in CSF circulating miRNAs have also been associated with different neurodegenerative diseases. For example, miRNA profiles can discriminate AD patients from healthy controls [61]. Analysing the same set of miRNAs that were studied in plasma, we show here for the first time a different miRNA profile in CSF of sheep naturally infected with scrapie. The levels of four miRNAs were significantly increased in scrapie CSF. The higher number of detected alterations could possibly be expected due to the direct interaction between CSF and brain extracellular space. Once again, miR-21-5p appeared to be upregulated in scrapie. miR-21-5p levels have been shown to be increased, among others, in CSF of acute encephalitis patients infected with Japanese Encephalitis virus [62], temporal lobe epilepsy [63] and glioma. Therefore, it must be considered that elevated levels of miR-21-5p in a wide range of pathologies involving CNS could diminish its usefulness as a specific biomarker for prion diseases. 

MicroRNA miR-342-3p, that was also upregulated in scrapie CSF, have also been found increased in encephalitis patients [62] but downregulated in plasma from AD patients [64]. The expression of this miRNA is also increased in brains from CJD patients and altered in murine models of prion disease, which may be a general phenomenon in the late stage of prion infection and might be used as a novel biomarker for animal and human TSEs [33,34]. Our studies, both in plasma and CSF, confirm the potentiality of this miRNA as scrapie biomarker. On the other hand, the levels of miR-146a-5p augmented in CSF of AD patients [64]. This miRNA, which is overexpressed in murine brain tissues infected with prions, seems to modulate microglial activation state [34,36]. However, it has also been shown to play a role on inflammation, myeloid cell proliferation and oncogenic transformation [65]. In addition, high levels of miR-128-3p have also been related with other neuropathologies like acute ischemic stroke [66]. This miRNA is released in exosomes from prion-infected neuronal cells [31] and targets ATG1 to activate autophagy in brain cells after ischemia reperfusion [67]. Finally, miR132-3p, which was amplified almost exclusively in scrapie sheep, regulates neuronal differentiation, maturation and function and participates in axon growth, neural migration and plasticity [68]. This miRNA is in significant decline in the CNS of Alzheimer’s patients in late courses of the disease [69] and in a patient with progressive supranuclear palsy, a tauopathy disorder [70]. On the contrary, a significant increase of miR-132 has been observed in a rat model of Parkinson’s disease [71]. An abnormal transcription of miR-132-3p is observed at early phases of infection of hippocampal CA1 neurons infected with prions [72]. Our results indicate that this miRNA may play a role in the pathogenesis of prion diseases in vivo.

Isolation of CSF-derived sEVs for further miRNA quantification did not promote miRNA detection by signal amplification. Only two miRNAs were amplified in levels sufficient for reliable quantification and no differences were detected between scrapie and control sEVs. Although the amount of sEVs in CSF was very low, they could be detected by TEM, showing the expected size. It is possible that the sEV isolation yield could be improved by concentration of CSF. Although it is difficult to compare levels of miRNAs in total CSF and CSF-derived sEVs, we used four times the volume of CSF used for RNA extraction to isolate sEVs. Thus, it appears that, at least for this set of miRNAs, the amount of free or protein-incorporated CSF miRNAs is much higher than EV-incorporated miRNAs. This is consistent with previously published RNAse protection assay data that indicate that many miRNAs, including miR-21, are primarily found outside the exosomal vesicles [73].

This study has several limitations. First, the study of miRNAs was focused on a small number of miRNAs selected based on the literature and our previous findings in plasma. Our results encourage further studies focused on whole miRNome in both plasma and CSF, and not only free miRNAs but also exosomal miRNAs. Unbiased genome-wide transcriptomics approach would avoid discarding any potential miRNAs of interest for the development of new diagnostic biomarkers. Second, in the present study, two methodologies were applied for exosome isolation. Although the protocols for plasma isolation are relatively well established, exosome extraction from CSF would benefit from methodological optimisation to facilitate their use for diagnostic purposes. 

Taking our results and those of others together, the altered miRNAs analysed in this work could be biomarkers of brain damage, but they are not specific of prion diseases. However, the analysis of this battery of miRNAs could help in scrapie diagnosis once the presence of the disease is suspected. Although the use of CSF is not a choice for scrapie diagnosis, our results could also contribute to CJD diagnosis. Our findings suggest that the presence of PrP^Sc^ in sEVs derived from CSF may represent a potential source of biomarkers for TSEs and may facilitate in vivo diagnosis, at least during the clinical stage of the disease. Further studies are required, involving the amplification of PrP^Sc^ also in total CSF to compare it with that of sEV-enriched fraction to elucidate the potential of CSF-derived sEVs to be feasible biomarkers, which in the end may contribute to providing useful alternatives for current TSE diagnosis methodology.

## 4. Materials and Methods

### 4.1. Animals and Sample Collection

A total of 58 sheep were used in this study. Most of them were female Rasa Aragonesa sheep, aged between 2 and 9 years and displaying the ARQ/ARQ genotype, which is the most frequently found genotype in scrapie-affected animals of this breed [74]. This group of animals included 13 negative control sheep, 6 animals at preclinical stage of scrapie disease, 33 at clinical stage and 6 at terminal stage. Tables S1–S4 show the identification, breed, sex, *PRNP* genotype, age, disease status and experimental use of each one of the animals studied. 

Plasma was obtained from 5 mL of blood, which was collected by jugular venipuncture from each animal. Blood was centrifuged for 10 min at 1300× *g* at room temperature (RT) to separate plasma, divided in aliquots and stored at −80 °C until analysis. A volume of approximately 15 mL of CSF was obtained from each sheep immediately after sacrifice, using a 10 mL syringe inserted into the cisterna magna and avoiding blood contamination. CSF was also aliquoted and stored at −80 °C until analysis.

### 4.2. Extracellular Vesicle Isolation from Plasma and CSF

Two commercial kits were used for sEV isolation from plasma: Total Exosome Isolation Kit (from plasma) (Invitrogen, Waltham, MA, USA) and miRCURY^TM^ Exosome Isolation Kit—Serum and plasma (Exiqon). The initial amount of plasma used for sEV isolation was 250 µL for the first kit and 500 µL for the second one. We followed the manufacturer’s instructions, with a final resuspension of sEV pellets in 100 µL of PBS (Invitrogen, Waltham, MA, USA) or 270 µL of Resuspension Buffer (Exiqon). sEVs isolated with the first kit were subjected to PMCA for PrP^Sc^ detection (n = 24), whereas those obtained with miRCURY^TM^ kit were used for further RNA extraction and miRNA profiling (n = 16).

Total Exosome Isolation Kit (from other body fluids) (Invitrogen) was used for sEV isolation from CSF for further PMCA analysis (n = 19), and miRCURY^TM^ Exosome Isolation Kit—Cells, Urine and CSF (Exiqon) was used to isolate sEVs for miRNA profiling (n = 12). Both kits used a starting sample of 1 mL CSF to precipitate sEVs, with a final resuspension step in 50–75 µL of PBS (Invitrogen) or 100 µL of Resuspension Buffer (Exiqon). In all cases, the purified sEV samples were stored at −20 °C until their use.

### 4.3. sEVs Characterisation

#### 4.3.1. Transmission Electron Microscopy 

sEV morphology was analysed in two sEV samples obtained from plasma and CSF. The morphology was evaluated by transmission electron microscopy (TEM) using the methodology previously described [75,76]. A volume of 5 µL of resuspended sEVs were fixed in 2.5% glutaraldehyde and then washed with deionised water. Samples were contrasted with 2% uranyl acetate, embedded in 0.13% methyl cellulose and 0.4% uranyl acetate, and then visualised using a Tecnai T20 microscope (FEI Company, Hillsboro, OR, USA), with a filament of LaB6. The voltage used during the visualisation was 200 KV, and acquiring images was performed with a CCD 2 K × 2 K Veleta model (Olympus, Tokyo, Japan).

#### 4.3.2. Dynamic Light Scattering

The size distribution of nanoparticles was evaluated using dynamic light scattering (DLS) assays as previously described [76]. An aliquot of 25 µL of sEV precipitate was diluted in PBS to a final volume of 500 µL and measured in the NanoBrook 90Plus PALS Particle Analyzer (Brookhaven Instruments Corporation, Holtsville, New York, USA). Samples were hit by a diode laser of 35 mW, which allowed discrimination of particle sizes between 0.3 nm and 6 µm.

### 4.4. PrP^Sc^ Determination by PMCA

Presence of PrP^Sc^ in sEVs isolated from plasma (n = 24: 17 scrapie-infected sheep at clinical stage, 5 at terminal stage and 2 negative controls) and CSF (n = 19: 8 scrapie-infected sheep at clinical stage, 6 at preclinical stage, 3 at terminal stage and 2 negative controls) was analysed by PMCA.

PMCA was performed by mixing 5 µL of the seed with 45 µL of substrate per well in a 96-well PCR microplate (Axygen Scientific, Corning, NY, USA). The PrP^C^ substrate was prepared from brain tissue (10% brain lysate) of uninfected transgenic Tg338 mice expressing ovine PrP^C^ in cold PMCA buffer (50 mM tris-HCl, pH 7.4, 5 mM EDTA, 300 mM NaCl, 1% Triton X-100). One teflon bead (diameter, 2.381 mm) was added to each well. Each sample was run in duplicate using 1/5, 1/10 and 1/50 dilutions in PMCA buffer. Amplification was performed in a modified sonicator (QSonica Q700), using a water recirculation system (39.5 °C) [46]. Microplates were then submitted to 96 cycles of 10 s sonication (75% power) followed by a 14 min and 50 s incubation period. After the first PMCA round, 5 µL of the reaction product were added to a new microplate containing 45 µL of fresh substrate and a new round of 96 cycles was performed. A total of 4 rounds of PMCA (24 h each) were performed in this study. To limit the cross-contamination risks that are linked to serial PMCA, lines of unseeded substrate were included on each microplate and procedures were performed in different rooms using dedicated material. A 10-fold dilution series (10^−3^ to 10^−9^ diluted) of brain homogenate from ARQ/ARQ ovine scrapie sheep was included on each PMCA run to check the amplification performance. Brain homogenates were prepared in PBS (10% weight/volume) from medulla oblongata.

On completion of the PMCA procedure, Dot blot was used to analyse the presence of PrP^Sc^ in each PMCA round. PMCA products (18 µL) were supplemented with 2 µL of 3% SDS and treated with PK (final concentration, 50 µg/mL) for 1 h at 37 °C. Digestion was stopped by adding an equal volume of Laemmli buffer and heating at 95 °C for 5 min. A 5-µL volume of sample was mixed with 25 µL of 1% SDS. The samples were then vacuum transferred onto a nitrocellulose membrane. The membrane was rinsed once with PBS (0.1% Tween 20) and incubated for 30 min in PBST containing 2% BSA. The monoclonal antibody (mAb) Sha31 (1:8000 dilution in PBST + 2% BSA), which recognises residues 145–152 (YEDRYYRE) of PrP, was used for PrP^Sc^/PrP^res^ immunodetection [77]. Antibody binding was detected by incubating the membranes for 20 min with conjugated mouse anti-goat IgG (1:5000 dilution; Santa Cruz Biotechnology, Dallas, TX, USA). Immunoblots were developed by enhanced chemiluminescence using ECL reagent (Pierce, Waltham, MA, USA) and visualised using the Versa Doc Quantity One image analysis system (Bio-Rad, Hercules, CA, USA).

TeSeE Western blot kit (Bio-Rad, Hercules, CA, USA) was used following the manufacturer’s recommendations to analyse the presence of PrP^Sc^ in the last round of each PMCA procedure. A volume of 20 µL of PMCA reaction product was mixed with 180 µL of 10% negative brain sheep homogenate before PrP^Sc^ extraction, as previously described [46]. PrP^Sc^ detection was performed using Sha31 mAb conjugated to horseradish peroxidase (0.06 µg/mL), and ECL substrate (Pierce) was used to reveal peroxidase activity.

### 4.5. Bioassay in Tg338 Mice

sEVs isolated from CSF using both commercial kits were stored at 4 °C and used as inocula for the bioassay in transgenic Tg338 mice within the next 48 h. Intracerebral inoculations were performed under gaseous anesthesia using 50 µL syringes and 25-gauge hypodermic needles inserted into the right parietal lobe, delivering 20 µL of the inoculum to each animal. Nine mice were inoculated with CSF-derived sEVs obtained with Exiqon kit, 9 mice with CSF-derived sEVs obtained with Invitrogen kit and 9 mice with total CSF. In each group, 3 mice were inoculated with CSF or CSF-derived sEVs from a negative control sheep and 6 mice with CSF or CSF-derived sEVs from two positive scrapie sheep. To reduce post-inoculation pain, a subcutaneous injection of buprenorphine (0.3 mg/kg) was administered to each mouse before recovery to consciousness. After inoculation, mice were housed in filtered cages and their clinical status was monitored three times a week.

#### Histopathological Analysis of Inoculated Mice

Mouse brains were fixed in formalin, embedded in paraffin, cut into 4 µm sections, and mounted on glass slides for histological evaluation using haematoxylin and eosin staining. The level of vacuolisation was evaluated in each sample using a semiquantitative score with a scale of 0 (lack of vacuolisation) to 5 (very high presence of vacuoles). On the other hand, an analysis of PrP^Sc^ deposition was performed using the paraffin-embedded tissue-blot (PET-blot) method as previously described [78]. Briefly, 4 μm paraffin-embedded brain sections were collected onto a nitrocellulose membrane and dried at 56 °C for 24 h. Afterwards, the membranes were subjected to dewaxing and rehydration and incubated for 2 h in a PK solution (250 μg/mL) at 56 °C to digest PrP^C^. The remaining PrP^Sc^ was denatured by incubating the membranes in a solution of guanidine thiocyanate 3 M. After blocking the membranes with 0.2% BSA, PrP^Sc^ was detected using the Sha31 mouse mAb (1:8000, SPI-Bio) and a secondary alkaline phosphatase-conjugated antibody (1:500, Agilent Dako). Enzymatic activity was visualised using NBT/BCIP (Thermo Scientific, Waltham, MA, USA).

### 4.6. RNA Purification and miRNA Quantification

#### 4.6.1. sEV RNA Extraction

After sEV precipitation from plasma using miRCURY^TM^ Exosome Isolation Kit (n = 16, 8 from healthy and 8 from scrapie-infected sheep), total sEV RNA extraction was carried out using miRCURY^TM^ RNA Exosome Isolation Kit-Biofluids (Exiqon) following the manufacturer’s recommendations. RNA from CSF-derived sEVs (n = 12, 6 from healthy and 6 from scrapie-infected sheep) was extracted using miRCURY^TM^ RNA Isolation Kit—Cell and Plant (Exiqon, Düsseldorf, Germany) according to the manufacturer’s recommendations. In both cases, precipitated sEVs were lysed with the appropriate volume of Lysis Solution and subsequently spiked in with a 5.7 µL of a carrier/spike solution containing 0.5 µg MS2 RNA and 25 fmol cel-miR-39 and at the end of the process, RNA was eluted to 100 µL Elution Buffer and stored at −80 °C for miRNA expression analysis.

#### 4.6.2. Total RNA Extraction from CSF

Total RNA was purified from 200 µL of CSF and 200 µL of CSF after sEV purification (sEV-depleted CSF) using Total RNA Purification Kit (Norgen, Thorold, ON, Canada) according to the manufacturer’s instructions. Briefly, the samples were lysed with 600 µL of Lysis Buffer and carrier/spike solution was added as above. After adding 600 µL ethanol the lysate was passed through RNA binding columns, the bound RNA was washed and eluted to 50 µL Elution Solution and stored at −80 °C for miRNA expression analysis.

#### 4.6.3. Retrotranscription and miRNA Amplification

Retrotranscription was performed using specific stem-loop TaqMan MicroRNA Assays (Applied Biosystems, Waltham, MA, USA) for each miRNA and TaqMan MicroRNA Reverse Transcription Kit (Applied Biosystems). For each sample, retrotranscription for seven miRNAs and cel-miR-39, used as a “spike-in” normaliser, was performed in a pooled reaction containing 9 μL of template RNA. The sequence for each miRNA is shown in Table S5. Individual miRNAs were quantified using the previously described methodology [32,35]. Briefly, qPCR was performed in three technical replicate reactions consisting of 2.5 μL 2× TaqMan Fast Universal PCR Master Mix, no AmpErase UNG (Life Technologies, Waltham, MA, USA), 0.25 μL TaqMan miRNA Assay probe (Life Technologies, Waltham, MA, USA) and 2.25 μL of complementary DNA diluted 1:5 in nuclease-free water. Cycling conditions were 95 °C for 20 s followed by 40 cycles of 95 °C for 1 s and 60 °C for 20 s. Sample/probe combinations where Ct values exceeded 34 cycles were omitted. For each sample, the mean Ct of technical replicates for each miRNA was normalised using the mean Ct of spiked-in cel-miR-39. Relative miRNA expression was determined with the 2^−∆∆Ct^ method [79] using healthy control sheep as calibrators. Differences between groups were statistically evaluated using Student’s *t*-test.

## 5. Conclusions

Analysis of circulating biofluids could facilitate and/or complement current diagnostic markers for prion disease as these fluids are known to harbour PrP^Sc^ and extracellular miRNAs. To what extent these potential biomarkers may associate with extracellular vesicle compartment in these biofluids remains largely unknown. Two of the most frequently studied biofluids in neurodegenerative conditions are CSF and blood plasma. We demonstrated that Prp^Sc^/PrP^res^ is present in CSF sEVs from scrapie sheep but was not detected in sEVs from plasma, despite the latter containing more sEVs. The CSF-derived, Prp^Sc^-containing sEVs did not demonstrate infectivity in vivo. Both plasma and CSF displayed some miRNA alterations in natural sheep scrapie, the CSF showing increased number of altered miRNA species. Purification of sEV fraction from the said biofluids did not lead to better detection levels of studied miRNAs, even if the amount of biofluids used for sEV extraction was considerably larger. Both the presence of PrP^Sc^ and the altered levels of several studied candidate miRNAs in CSF could facilitate the development of biofluid-based biomarkers for the prion diseases in the future.

## Data Availability

The raw data of the results presented in this study are available on request from the corresponding author.

## Supplementary Materials

The following are available online at https://www.mdpi.com/article/10.3390/ijms22136822/s1.
