# Supplementary material for: Cerebrospinal Fluid and Plasma Small Extracellular Vesicles and miRNAs as Biomarkers for Prion Diseases
Source: Int J Mol Sci. 2021 Jun 25;22(13):6822. doi: 10.3390/ijms22136822 (PMC8268953; doi:10.3390/ijms22136822)
Supplement: Supplementary file 1 [file ijms-22-06822-s001.zip › ijms-1233242-supplementary.pdf]

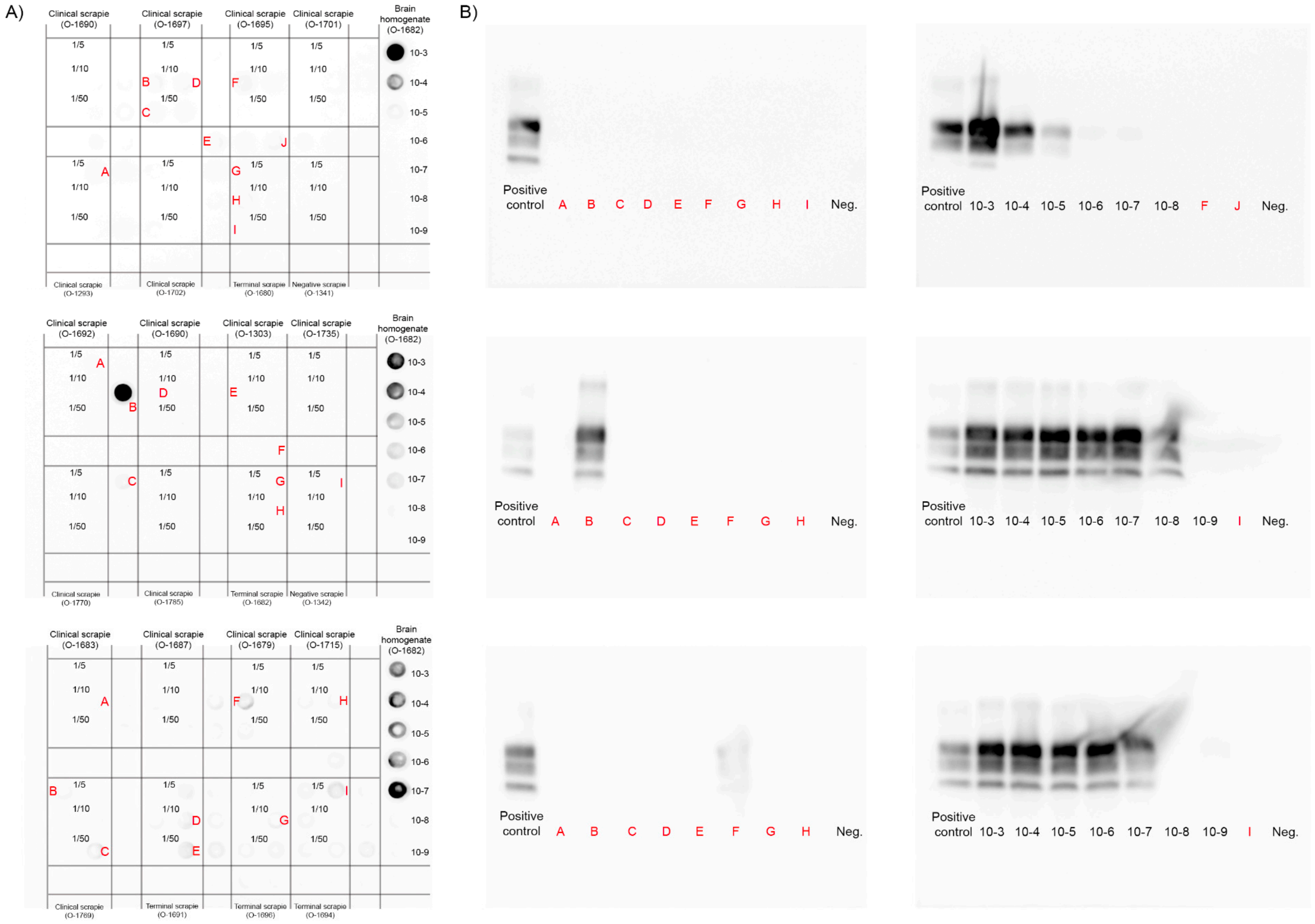

Figure S1. Detection of the proteinase K-resistant core fragment (PrP^{res}) of the pathologic prion by Dot blot (A) and Western blot (B) in PMCA reactions seeded with serially diluted (1/5, 1/10 and 1/50) plasma-derived sEVs obtained from scrapie-affected sheep. Immunodetection was performed using the monoclonal Sha31 antibody. (A) PrP^{res} detection by Dot blot after 4 PMCA rounds of plasma-derived sEVs from sheep at clinical and terminal stage of scrapie and negative sheep. A brain homogenate from scrapie sheep (10^{-3} to 10^{-9} dilution) was included for comparison. Some dots (labelled in red letters) were subjected to Western blot for PrP^{res} profile detection. (B) PrP^{res} detection by Western blot after 4 PMCA rounds of plasma-derived sEVs, including the brain homogenate from scrapie sheep. A proteinase K-digested classical scrapie isolate (Dawson strain) was used as positive control. No PrP^{res} was detected in plasma-derived sEVs. However, positivity was detected in one unseeded well (middle membrane, B letter) and one seeded well (bottom membrane, F letter). The Western blot PrP^{res} pattern (glycoprofile and mobility) in these positive reactions was typical of scrapie prion and identical to that observed in the brain homogenate. The experiment was repeated for the positive seeded well, and no PrP^{res} was detected after 4 PMCA rounds (see Figure S2), making a cross contamination between wells a likely explanation.

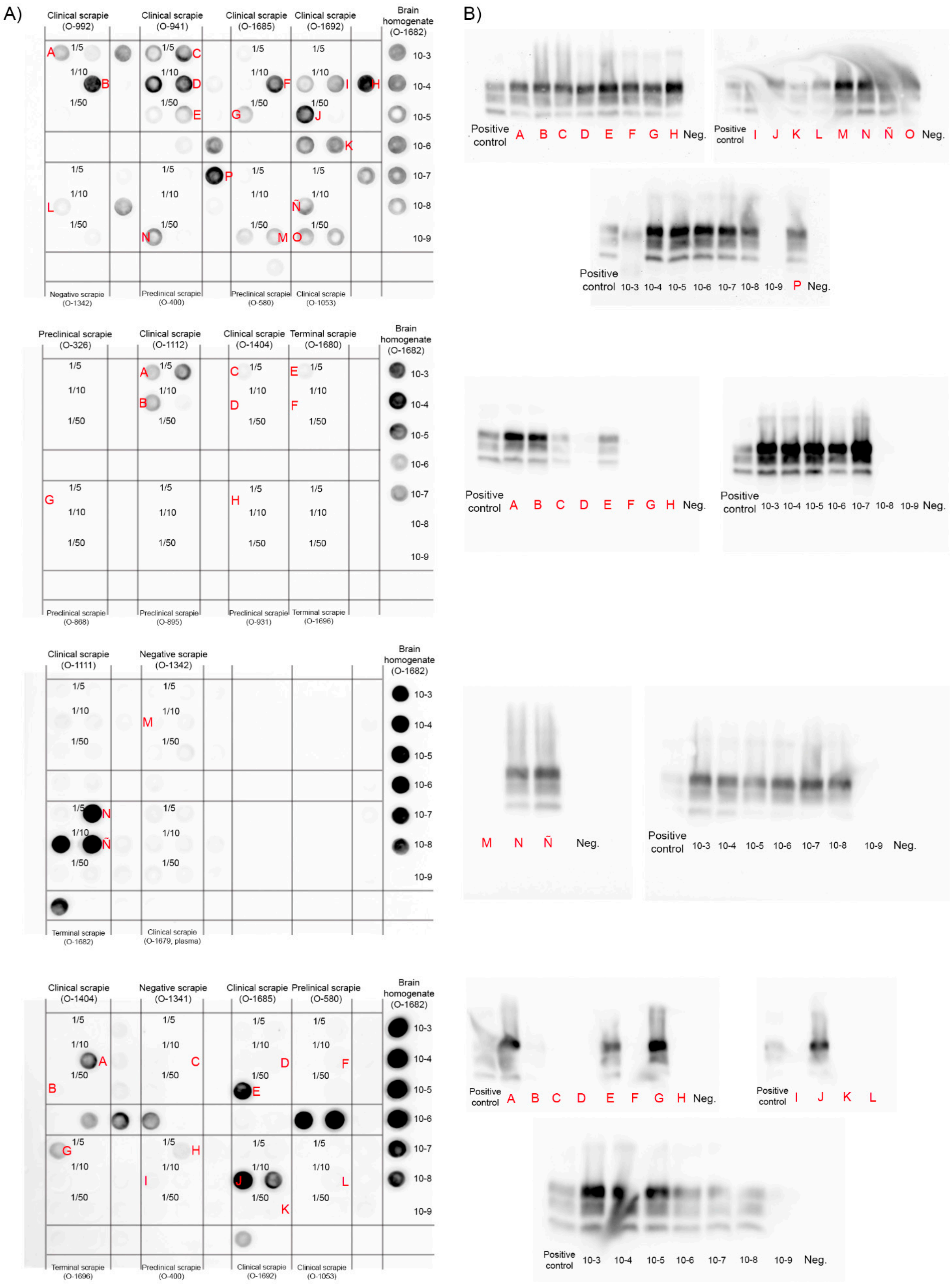

Figure S2. Detection of the proteinase K-resistant core fragment (PrPres) of the pathologic prion by Dot blot (A) and Western blot (B) in PMCA reactions seeded with serially diluted (1/5, 1/10 and 1/50) CSF-derived sEVs obtained from scrapie-affected sheep. Immunodetection was performed using the monoclonal Sha31 antibody. (A) PrPres detection by Dot blot after 4 PMCA rounds of CSF-derived sEVs from sheep at preclinical, clinical and terminal stage of scrapie and negative sheep. A brain homogenate from scrapie sheep (10^{-3} to 10^{-9} dilution) was included for comparison. Some dots (labelled in red letters) were subjected to Western blot for PrPres profile detection. (B) PrPres detection by Western blot after 4 PMCA rounds of CSF-derived sEVs, including the brain homogenate from scrapie sheep. A proteinase K-digested classical scrapie isolate (Dawson strain) was used as positive control.

PrP^{res} was detected in reactions seeded with CSF-derived sEVs in 6 out of 8 samples from clinically-affected sheep (O-992, 941, 1685, 1692, 1112 and 1404) and 3 out of 3 samples from sheep at terminal stage (O-1680, 1682 and 1696). PrP^{res} was detected only in 1 out of 6 sEV samples obtained from CSF of preclinical scrapie sheep (O-580). In this preclinical animal we repeated the PMCA reaction three times to rule out cross-contamination or a false positive result. No PrP^{res} signal was detected in reactions seeded with CSF-derived sEVs from the negative control sheep (O-1341 and 1342). In addition, one sample of plasma-derived sEVs (O-1679) was repeated and no PrP^{res} was detected.

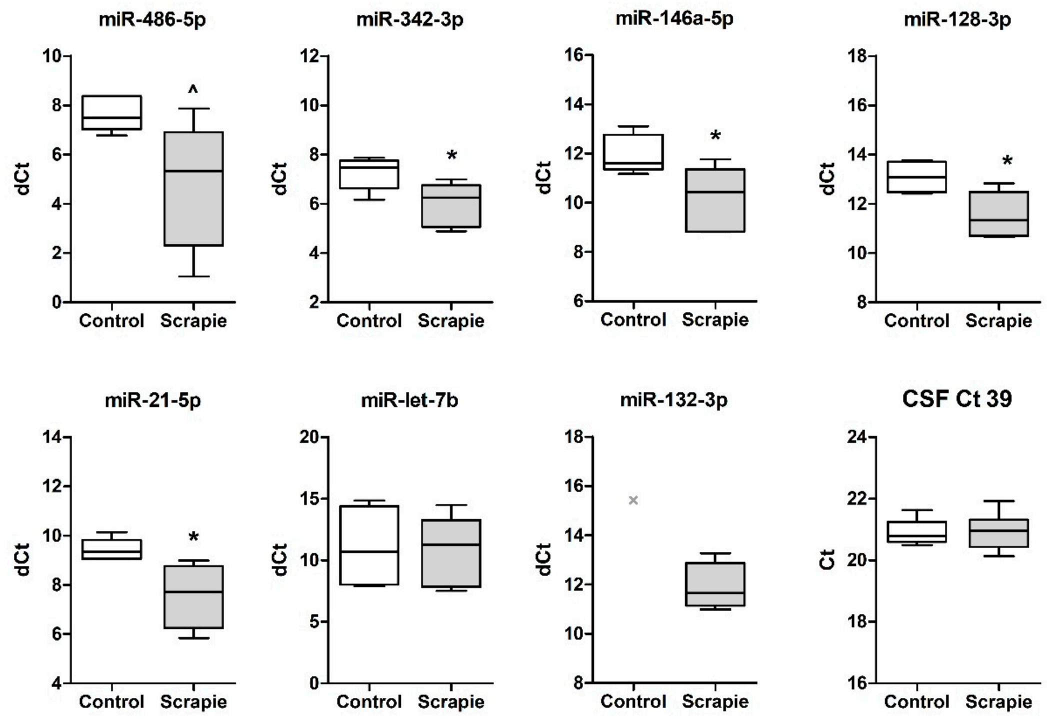

Figure S3. Quantification of miRNAs in CSF by qPCR. The data presented in Figure 5A are shown as the difference in the Ct values between the spike in normalizer cel-miR-39 and the measured miRNA. The error bars of the box plots indicate the minimum and maximum values for the sheep in each experimental group. In the case of miR-132-3p, only one control sheep expressed this miRNA in detectable levels. The CSF Ct 39 indicates that the Ct values of the spike in normalizer between the experimental groups was not different (P = 0.937). Statistical significance was assessed by Student's t-test (*P < 0.05, ^P < 0.01). In the case of miR-132-3p the statistical test was not possible.

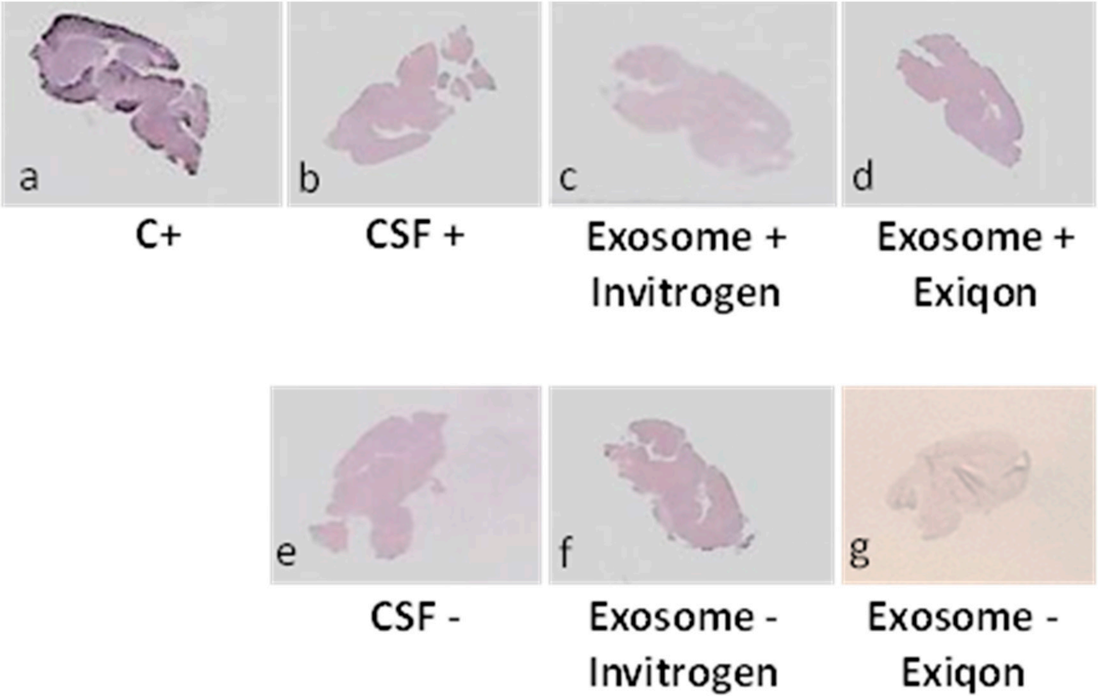

Figure S4. PET-Blot analysis of Tg338 inoculated mice: (a) Positive control, (b) mouse brain inoculated with CSF from positive scrapie sheep, (c) mouse brain inoculated with CSF-derived sEVs from positive scrapie sheep obtained with Invitrogen kit, (d) mouse brain inoculated with CSF-derived sEVs from positive scrapie sheep obtained with Exiqon miRCURY kit, (e) mouse brain inoculated with CSF from negative scrapie sheep, (f) mouse brain inoculated with CSF-derived sEVs from negative scrapie sheep obtained with Invitrogen kit and (g) mouse brain inoculated with CSF-derived sEVs from negative scrapie sheep obtained with Exiqon miRCURY kit.

Table S1: Sheep used in PMCA of plasma-derived sEVs. Table includes animal ID, breed, sex, *PRNP* genotype, age (years) and disease status.

Animal ID	Breed	Sex	*PRNP* genotype	Age (years)	Disease status
**O-1341**	Mixed	Female	AHQ/VRQ	3	Negative
**O-1342**	Mixed	Female	ARQ/VRQ	3	Negative
**O-1293**	Mixed	Male	VRQ/VRQ	4	Clinical
**O-1303**	Rasa Aragonesa	Female	ARQ/ARQ	4	Clinical
**O-1679**	Rasa Aragonesa	Female	ARQ/ARQ	5	Clinical
**O-1683**	Rasa Aragonesa	Female	ARQ/ARH	2	Clinical
**O-1690**	Rasa Aragonesa	Female	ARQ/ARQ	5	Clinical
**O-1690**	Rasa Aragonesa	Female	ARQ/ARQ	5	Clinical
**O-1692**	Rasa Aragonesa	Female	ARQ/ARQ	8	Clinical
**O-1695**	Rasa Aragonesa	Female	ARQ/ARQ	4	Clinical
**O-1687**	Rasa Aragonesa	Female	ARQ/ARQ	3	Clinical
**O-1697**	Rasa Aragonesa	Female	ARQ/ARQ	3	Clinical
**O-1701**	Rasa Aragonesa	Female	ARQ/ARH	2	Clinical
**O-1702**	Rasa Aragonesa	Female	ARQ/ARQ	3	Clinical
**O-1715**	Rasa Aragonesa	Female	ARQ/ARQ	4	Clinical
**O-1735**	Rasa Aragonesa	Female	ARQ/ARQ	9	Clinical
**O-1769**	Rasa Aragonesa	Female	ARQ/ARQ	5	Clinical
**O-1770**	Rasa Aragonesa	Female	ARQ/ARQ	5	Clinical
**O-1785**	Rasa Aragonesa	Female	ARQ/ARQ	4	Clinical
**O-1680**	Rasa Aragonesa	Female	ARQ/ARH	2	Terminal
**O-1682**	Rasa Aragonesa	Female	ARQ/ARQ	4	Terminal
**O-1691**	Rasa Aragonesa	Female	ARQ/ARQ	5	Terminal

**O-1694**	Rasa Aragonesa	Female	ARQ/ARQ	5	Terminal
**O-1696**	Rasa Aragonesa	Female	ARQ/ARH	2	Terminal

Table S2: Sheep used in PMCA of CSF-derived sEVs. Table includes animal ID, breed, sex, *PRNP* genotype, age (years) and disease status.

**Animal ID**	**Breed**	**Sex**	***PRNP* genotype**	**Age (years)**	**Disease status**
**O-1341**	Mixed	Female	AHQ/VRQ	3	Negative
**O-1342**	Mixed	Female	ARQ/VRQ	3	Negative
**O-326**	Rasa Aragonesa	Female	ARQ/ARQ$_{141}$L/F	5	Preclinical
**O-580**	Rasa Aragonesa	Female	ARQ/ARQ	3	Preclinical
**O-400**	Rasa Aragonesa	Female	ARQ/ARQ	4	Preclinical
**O-868**	Rasa Aragonesa	Female	ARQ/ARQ	2	Preclinical
**O-895**	Rasa Aragonesa	Female	ARQ/ARQ	4	Preclinical
**O-931**	Rasa Aragonesa	Female	ARQ/ARQ	1	Preclinical
**O-992**	Rasa Aragonesa	Female	ARQ/ARQ	7	Clinical
**O-941**	Rasa Aragonesa	Female	ARQ/ARQ	4	Clinical
**O-1053**	Rasa Aragonesa	Female	ARQ/ARQ	7	Clinical
**O-1111**	Rasa Aragonesa	Female	ARQ/ARQ$_{143}$H/R	3	Clinical
**O-1112**	Rasa Aragonesa	Female	ARQ/ARQ	3	Clinical
**O-1404**	Rasa Aragonesa	Female	VRQ/VRQ	4	Clinical
**O-1685**	Rasa Aragonesa	Female	ARQ/ARQ	2	Clinical
**O-1692**	Rasa Aragonesa	Female	ARQ/ARQ	8	Clinical
**O-1680**	Rasa Aragonesa	Female	ARQ/ARH	2	Terminal
**O-1682**	Rasa Aragonesa	Female	ARQ/ARQ	4	Terminal
**O-1696**	Rasa Aragonesa	Female	ARQ/ARH	2	Terminal

Table S3: Sheep used in miRNAs analysis of plasma-derived sEVs. Table includes animal ID, breed, sex, *PRNP* genotype, age (years) and disease status.

**Animal ID**	**Breed**	**Sex**	***PRNP* genotype**	**Age (years)**	**Disease status**
**O-1761**	Rasa Aragonesa	Female	Non-genotyped	8	Negative
**O-1762**	Rasa Aragonesa	Female	Non-genotyped	7	Negative
**O-1751**	Rasa Aragonesa	Female	Non-genotyped	8	Negative
**O-1752**	Rasa Aragonesa	Female	Non-genotyped	8	Negative
**O-1757**	Rasa Aragonesa	Female	Non-genotyped	6	Negative
**O-1763**	Rasa Aragonesa	Female	Non-genotyped	8	Negative
**O-1756**	Rasa Aragonesa	Female	Non-genotyped	8	Negative
**O-1758**	Rasa Aragonesa	Female	Non-genotyped	7	Negative
**O-1695**	Rasa Aragonesa	Female	ARQ/ARQ	4	Clinical
**O-1702**	Rasa Aragonesa	Female	ARQ/ARQ	3	Clinical
**O-1685**	Rasa Aragonesa	Female	ARQ/ARQ	2	Clinical
**O-1667**	Rasa Aragonesa	Female	ARQ/ARQ	2	Clinical
**O-1669**	Rasa Aragonesa	Female	ARQ/ARQ	5	Clinical
**O-1694**	Rasa Aragonesa	Female	ARQ/ARQ	5	Terminal
**O-1684**	Rasa Aragonesa	Female	ARQ/ARQ	2	Terminal
**O-1681**	Rasa Aragonesa	Female	ARQ/ARQ	2	Terminal

Table S4: Sheep used in miRNAs analysis of CSF-derived sEVs. Table includes animal ID, breed, sex, *PRNP* genotype, age (years) and disease status.

Animal ID	Breed	Sex	*PRNP* genotype	Age (years)	Disease status
**O-1753**	Rasa Aragonesa	Female	Non-genotyped	8	Negative
**O-1760**	Rasa Aragonesa	Female	Non-genotyped	8	Negative
**O-1761**	Rasa Aragonesa	Female	Non-genotyped	8	Negative
**O-1762**	Rasa Aragonesa	Female	Non-genotyped	7	Negative
**O-1751**	Rasa Aragonesa	Female	Non-genotyped	8	Negative
**O-1754**	Rasa Aragonesa	Female	Non-genotyped	6	Negative
**O-647**	Rasa Aragonesa	Female	ARQ/ARQ	8	Clinical
**O-508**	Rasa Aragonesa	Female	ARQ/ARQ$_{143}$H/R	13	Clinical
**O-201**	Rasa Aragonesa	Female	ARQ/ARQ	5	Clinical
**O-602**	Rasa Aragonesa	Female	ARQ/ARQ	6	Clinical
**O-520**	Rasa Aragonesa	Female	ARQ/ARQ	5	Clinical
**O-673**	Ojinegra	Female	ARQ/ARQ	6	Terminal

Table S5: List of miRNA assays used. "Assay ID" and "Assay name" are as described by the provider of the miRNA assays (Life Technologies). "miRNA detected" depicts the arm (-5p or -3p) of the mature miRNA detected and the sequence for the corresponding miRNA is shown in the last column.

Assay ID	Assay name	miRNA detected	Mature miRNA sequence
000397	hsa-miR-21	miR-21-5p	UAGCUUAUCAGACUGAUGUUGA
006215_mat	xtr-miR-146	miR-146a-5p	UGAGAACUGAAUUCCAUAGGUU
002365	hsa-miR-494-3p	miR-494	UGAAACAUACACGGGAAACCUC
002221	hsa-let-7i-5p	let-7i	UGAGGUAGUAGUUUGUGCUGUU
000382	hsa-let-7f-5p	let-7f	UGAGGUAGUAGAUUGUAUAGUU
476665_mat	chi-miR-342-3p	miR-342-3p	UCUCACACAGAAAUCGCACCCA
473715_mat	chi-miR-342-5p	miR-342-5p	AGGGGUGCUAUCUGUGGUUGAGG
002619	hsa-let-7b	let-7b-5p	UGAGGUAGUAGGUUGUGUGGUU
002283	hsa-let-7d	let-7d-5p	AGAGGUAGUAGGUUGCAUAGUU
001278	hsa-miR-486-5p	miR-486-5p	UCCUGUACUGAGCUGCCCCGAG
002216	hsa-miR-128a	miR-128-3p	UCACAGUGAACCGGUCUCUUU
00200	cel-miR-39	cel-miR-39-3p	UCACCGGGUGUAAAUCAGCUUG
